# An automatic system for determining the effects of temperature on the hysteresis curves of ion-selective electrodes

**DOI:** 10.1155/S1463924697000060

**Published:** 1997

**Authors:** Júlia M. C. S. Magalhães, Adélio A. S. C. Machado

**Affiliations:** LAQUIPAI Faculdade de Ciências R. Campo Alegre 687 Porto P4150 Portugal

## Abstract

This paper describes an automatic system which measures the effect of temperature variations on the response of ion-selective electrodes (hysteresis curves). The system is managed by a computer program which plots hysteresis curves following a pre-established temperature cycle, from setting and controlling the temperature of the water-bath, to acquiring the response potentials of up to five electrodes after temperature stabilization.


Journal of Automatic Chemistry, Vol. 19, No. 2 (March–April 1997) pp. 39–44

### An automatic system for determining the effects of temperature on the hysteresis curves of ion-selective electrodes

**Júlia M. C. S. Magalhães and Adélio A. S. C. Machado***

*LAQUIPAI, Faculdade de Ciências, R. Campo Alegre 687, P4150 Porto, Portugal*

*This paper describes an automatic system which measures the effect of temperature variations on the response of ion-selective electrodes (hysteresis curves). The system is managed by a computer program which plots hysteresis curves following a pre-established temperature cycle, from setting and controlling the temperature of the water-bath, to acquiring the response potentials of up to five electrodes after temperature stabilization.*

### Introduction

The pH sensitive glass electrodes on the market have an internal reference solution with a fixed composition in order to place the concentration of the isopotential point in the middle of the operational range of measured values, thus minimizing the effects of the temperature variation on the electrode response [1, 2]. There has seemingly been little effort made to study the effect of the temperature on the response of other ion-selective electrodes in order to improve their performance. The early literature on ion-selective electrodes contains only a few papers on the subject, published in the early 1970s [3–5]. Work at the authors' laboratory on minimizing the effect of temperature on the response of ion-selective electrodes by adjusting the position of the isopotential point [6], then showed that for a PVC membrane calcium sensitive electrode with internal reference solution, the concentration of the isopotential point could be adjusted by varying the solution's composition [7].

The possibility of optimizing the construction procedure of all-solid-state ion-selective electrodes to bring the concentration of the isopotential point to the range of operational concentration values was also investigated, both for plastic [7–9] and crystalline [10–12] membrane electrodes. Temperature effects on all-solid-state electrodes are complex, because the devices involve several materials with different thermal properties and a badly defined internal reference. The results obtained showed that the effect of the temperature variation on the response of all-solid-state ion-selective electrodes depends on the nature and composition of the conductive support used, its internal electric contact and the type of membrane [6]. No systematic relations were found between these construction parameters of the electrodes and the coordinates of their isopotential points, so the optimization of the construction procedure of all-solid-state electrodes has to be trial-and-error [5, 6], involving a large amount of experimental work and time-consuming experiments. A hysteresis experiment for all-solid-state ion-selective electrodes involving cycles of five temperature values ($10 \rightarrow 50 \rightarrow 10\,^{\circ}C$ in $10\,^{\circ}C$ steps), for example, typically requires seven to eight hours of practically continuous monitoring of temperature and the corresponding response potentials of the electrodes [10]. Therefore, it was decided to assemble a system for automatic handling of experiments to determine the temperature hysteresis curves of ion-selective electrodes in isothermal cells (indicator and reference electrodes both immersed in the same cell, the temperature of which is varied) [13]. In favourable situations, these curves provide full information about the temperature behaviour of the electrodes, including the position of the isopotential point [14]. The assembly of the system, the software for its operation, and the results obtained when it was used to plot hysteresis curves for all-solid-state PVC membrane ammonium electrodes with nonactin as sensor, are described in this paper.

* Correspondence to A. Machado.

### Description of the system

### *Assembly of the system*

The automatic system, shown in figure 1, consists of a Techne thermoregulator, model TU-16D, in a water-bath of 5 l capacity; a Metrohm thermostated double-wall cell with 50 ml of capacity; a home-made high impedance circuit box, which allows parallel or, rather, almost simultaneous successive readings of differences of potential for up to five electrodes, against the same reference electrode; a Compaq Prolinea 325s, PC with a Lab Master DMA card (Scientific Solutions Inc.).

The water-bath temperature is controlled by the PC. The temperature is set by an analogue signal generated, via software, by the A/D converter of the Lab Master DMA card, and sent to the thermoregulator. An analogue signal sent by the thermoregulator to the A/D converter of the card is used to monitor the temperature. The response potentials of the electrodes are also acquired by the A/D converter of the Lab Master DMA card through the high impedance circuit box.

### *Control software*

The control software was developed in Quick Basic, version 4.5 (Microsoft). The control subroutines in the

0142-0453/97 $12·00 © 1997 Taylor & Francis Ltd

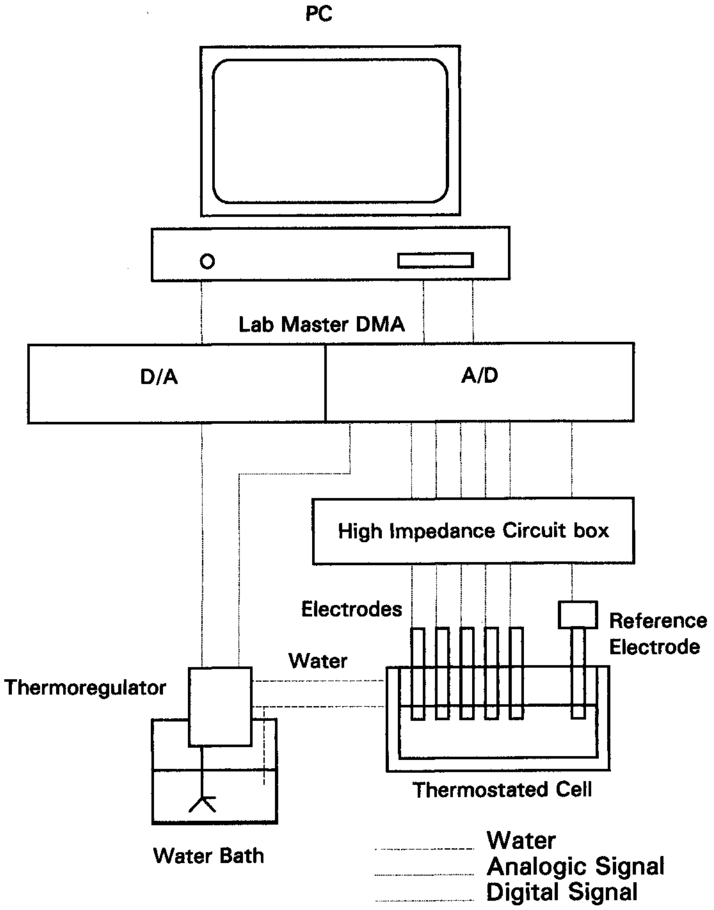

*Figure 1. Schematic representation of the system.*

card allow the interaction with the card via software in Quick Basic. The program involves:

(1) Reading the conditions for the execution of the experiment, including the number of different temperatures to be assayed (NT) and their set values (STi, i = 1, ..., NT), the criterion for stabilization of the water-bath before acquisition of the response potentials at each temperature (DT), the time for monitoring the response of the electrodes at each temperature (tMi), and the number of electrodes to be tested in parallel (Nel).

(2) Upon receiving this information, the program opens a set of 2 + Nel files to store results. A file is created for the time values at which the readings are taken (tk); another file contains temperature readings, Ti(tk); finally, a file is opened for the response potential readings for each electrode (Ej(tk), j = 1, ..., Nel).

(3) Execution and control of the experiment: this consists of the repetition of a cycle (cycle #1 in figure 2) for each temperature STi until all the temperatures are covered (i = NT). This cycle contains two interconnected subcycles: the first corresponds to the initial stabilization of the temperature (cycle #2); the second (cycle #3) is repeated along the time fixed for monitorization until this is exhausted. Cycle #3 includes: (*a*) comparison of the time elapsed from the beginning of the monitoring at each temperature (tk) with a pre-fixed time limit for monitoring (tMi) and ending the experiment if tk > tMi; (*b*) acquisition of the response potentials of the electrodes, Ej(tk), j = 1, ... Nel; (*c*) acquisition of the value of the temperature of the water-bath, Ti(tk); (*d*) recording the values of tk, Ti(tk) and Ej(tk), in the relevant files. The recorded time refers to the moment between the reading of potentials and the reading of temperature, the difference of these two operations being less than 0·2 s. The values stored in the files are simultaneously shown in the PC screen.

(4) The execution of the program is concluded by closing the files with acquired data, they are then transferred to an Excel worksheet where the data are treated and the results are plotted and printed out.

The A/D converter allows 40,000 potential measurements per second to be read. In the present application, and for noise reduction purposes, the program averages 500 readings per point. This number of readings allows response potentials with standard deviations lower than ±0·05 mV to be acquired. Averaging 500 readings per point corresponds to 80 potential readings per second, changes in the response potentials of up to five electrodes can be followed. Therefore, the high impedance circuit box was built for this number of electrodes.

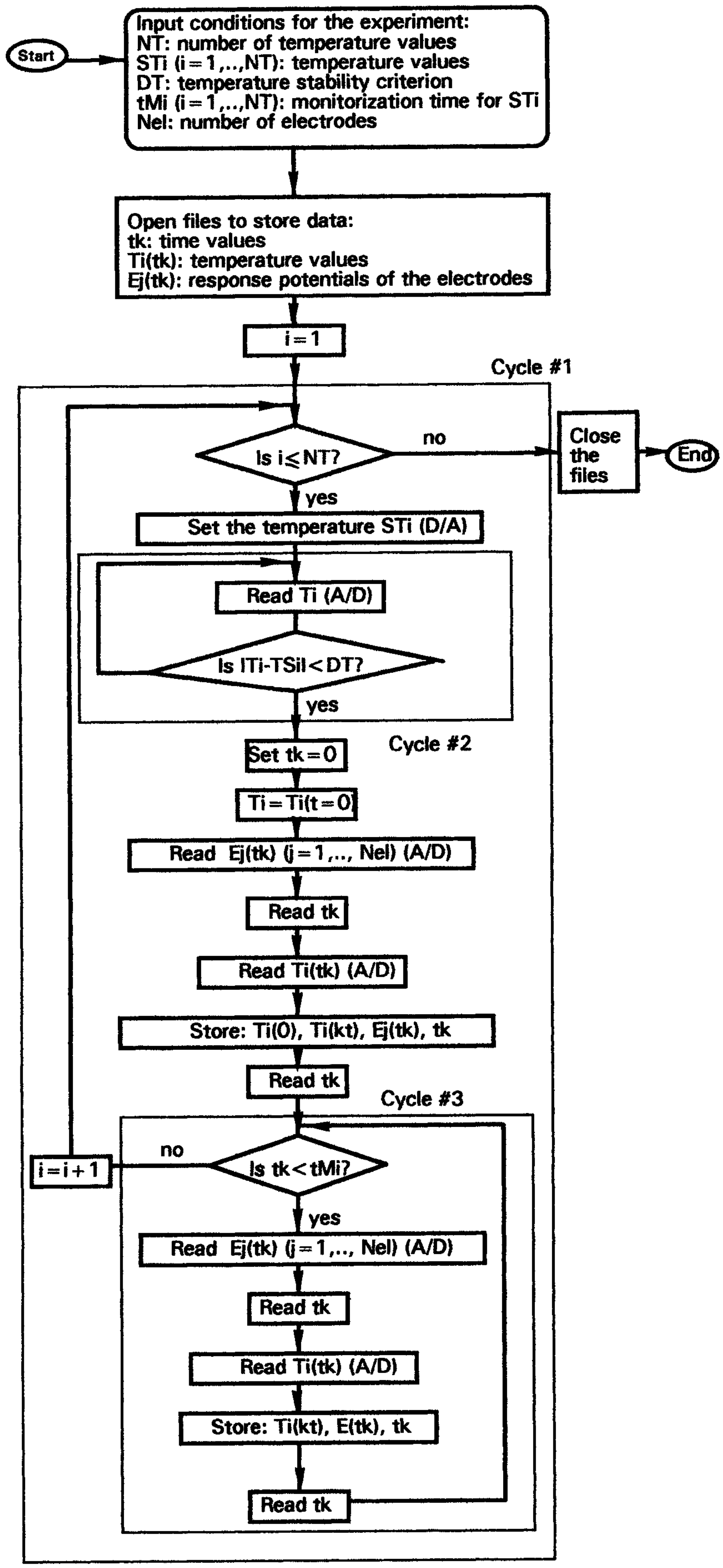

*Figure 2. Flow-chart of the program controlling the system (D/A and A/D indicate operation of the LabMaster DMA card through instructions in Quick Basic).*

### Experimental

### *Evaluation of the performance of the system*

Precision of the control of the water-bath temperature was evaluated by monitoring temperature for periods of about 11 consecutive hours; there was a one minute interval between consecutive readings (600–700 readings). The time required for the water-bath to stabilize at final temperatures of up to 55 °C, after temperature jumps with amplitudes between 4 to 37 °C, was also evaluated.

The global performance of the system was evaluated by monitoring the variation with temperature of the response potentials of ammonium ion-selective electrodes to obtain hysteresis curves. The electrodes were immersed in solutions of ammonium chloride of constant concentration and the temperature was varied from 10 to 40 °C and back again to 10 °C, in 10 °C steps.

### *Electrodes and reagents*

All-solid-state nonactin ammonium selective electrodes were prepared by casting a PVC membrane, 1 mm thick, on a graphite/epoxy conductive support [6, 15]. The solution used for preparing the sensing membrane of the ammonium electrode consisted of 3% of nonactin (Fluka, Ammonium Ionophore I), 33% of PVC (Fluka), and 64% of dibutylsebacate (Fluka, Selectophore), all dissolved in THF (Merck). The graphite powder used for the preparation of the conductive support was from Merck (<50 mm). The epoxy resin used to bind the graphite in the conductive support was HUNF, from Epoxy Technology, Inc. (1 : 1 graphite to epoxy weight proportion). When not in use, the ammonium electrodes were conditioned in a 0·1M ammonium chloride solution.

Reference electrodes were Ag/AgCl electrodes (double junction, Orion 90-02-00). These were used because they have low temperature hysteresis, large operational temperature ranges and short stabilization times [10, 16, 17].

### Results and discussion

### *Control of the water-bath temperature*

Figure 3 shows the temperature values stored during about 11 h of monitoring, when the water-bath control set was fixed, respectively to 25 and 45 °C. No drift of temperature was observed and the difference between the maximum and minimum values of temperature was lower than 0·2 °C in both cases. Further results obtained in the evaluation of the response of the system to temperature changes, and of the precision of the temperature attained after stabilization, are summarized

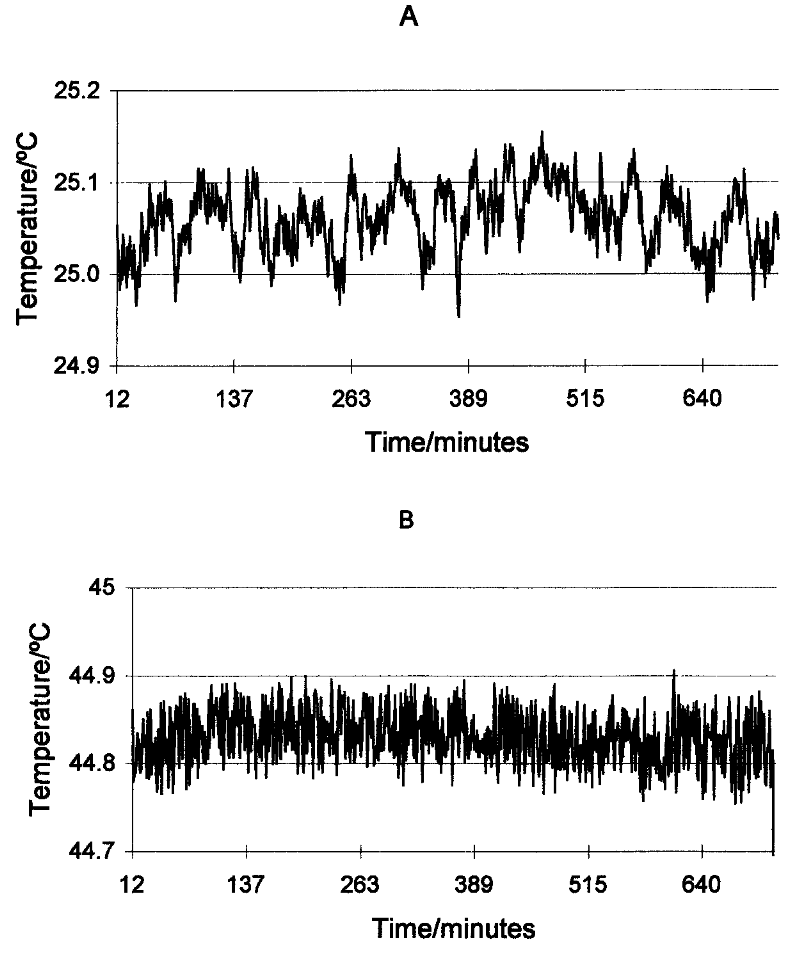

*Figure 3. Values of the temperature of the water-bath measured over about 11 h: (a) 25 °C and (b) 45 °C.*

*Table 1. Stability of the temperature of the water-bath.*

| Set | Temperature/°C Initial | Final[1] | Stabilization time[2]/min |
|---|---|---|---|
| 25 | 17·5 | 25·01 ± 0·03 | 3[3] |
| | 21·0 | 25·02 ± 0·04 | 3 |
| | 20·3 | 25·02 ± 0·05 | 3 |
| 35 | 24·0 | 34·71 ± 0·05 | 4 |
| | 25·0 | 34·69 ± 0·05 | 4 |
| | 25·5 | 34·68 ± 0·05 | 4 |
| 45 | 20·3 | 44·83 ± 0·03 | 7 |
| | 23·0 | 44·89 ± 0·04 | 7[4] |
| | 17·5 | 44·83 ± 0·03 | 8 |
| 55 | 45·0 | 55·10 ± 0·04 | 4 |
| | 20·3 | 55·08 ± 0·04 | 10 |
| | 18·5 | 55·08 ± 0·04 | 12 |

(1) Average and standard deviation calculated for 631 data points collected after temperature stabilization of the water-bath.

(2) Time before temperature stabilization.

(3) Experiment *a* in figure 3.

(4) Experiment *b* in figure 3.

in table 1. The standard deviations of the stabilized temperatures were less than ±0·05 °C for all experiments.

The times required for stabilization of the temperature of the bath show that thermal stability was reached in 3–12 min, depending on the difference between the initial and the set temperatures and on the direction of temperature change.

In conclusion, the system allows the temperature of the water-bath to be controlled with sufficient stability and precision for the application.

### *Hysteresis experiments*

Typical results obtained in a hysteresis experiment for an ammonium selective electrode in 0·5 mM ammonium chloride are presented in figure 4. Figure 4(*a*) shows the hysteresis curve and figure 4(*b*) shows the response potential of the electrode versus time, obtained at the different temperatures. These last plots allow the time at which the stabilization of response is attained to be identified and the corresponding average response potentials after stabilization at each temperature, which are used to plot the hysteresis curve, to be calculated.

To obtain a response hysteresis curve of the type shown in figure 4(*a*), the system requires approximately 150 min. However, the operator is only involved for 30 min, most of this time is used for preparation and for assembling the electrodes in the potentiometric cell.

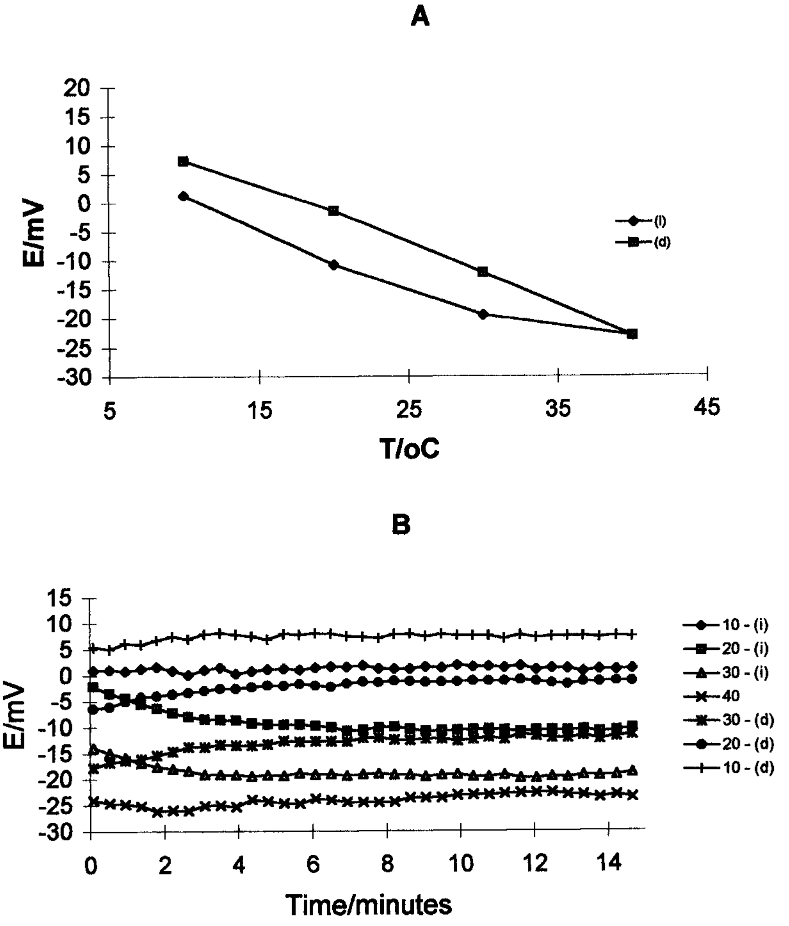

*Figure 4. Results of a hysteresis experiment with an ammonium selective electrode in 0·5 mM ammonium chloride (10 ° → 40 ° → 10°C in 10°C steps): (a) hysteresis curve; (b) variation of response with time at each temperature as indicated on the right-hand side: (i) increasing and (d) decreasing temperature.*

### Conclusions

The results obtained show that the system described is adequate for studying hysteresis due to temperature variations in the response of ion-selective electrodes. The system reduces the time needed for obtaining a hysteresis curve by about a half. In addition operator time is reduced by 90% compared with manual operation of a classical potentiometric system. The system can also be adapted for automatic calibration of a set of electrodes at several temperatures by connection to an automatic burette.

### Acknowledgements

Financial support received from JNICT, Lisbon, under Project PRAXIS XXI Nr 2/2.1/QUI/294/94, is gratefully acknowledged.

### References

1. Westcott, C. C., *pH Measurements* (Academic Press, New York, 1978), 14 and 25.
2. Midgley, D., *Analyst*, **112** (1989), 573 and 581.
3. Light, T. S. and Swartz, J. L., *Analytical Letters*, **1** (1968), 895.
4. Negus, L. E. and Light, T. S., *Instrumentation Technology*, **19** (1972), 23.
5. Vasconcelos, M. T. S. D. and Machado, A. A. S. C., *Port. Electrochim. Acta*, **7** (1989), 619.
6. Machado, A. A. S. C., *Analyst*, **119** (1994), 2263.
7. Vasconcelos, M. T. S. D. and Machado, A. A. S. C., *Analyst*, **115** (1990), 195.
8. Vasconcelos, M. T. S. D. and Machado, A. A. S. C., *Journal of Electroanalytical Chemistry*, **294** (1990), 209.
9. Vasconcelos, M. T. S. D. and Machado, A. A. S. C., *In Vivo Chemical Sensors: Recent Developments*, Eds. S. Alckock and A. P. F. Turner (Cranfield Press, Bedford, 1993), 120.
10. Vasconcelos, M. T. S. D. and Machado, A. A. S. C., *Analyst*, **113** (1988), 49.
11. Vasconcelos, M. T. S. D., Machado, A. A. S. C. and Rey, F., *Port. Electrochim. Acta*, **6** (1988), 167.
12. Vasconcelos, M. T. S. D. and Machado, A. A. S. C., *Journal of Electroanalytical Chemistry*, **279** (1990), 43.
13. Bethune, A. J., Light, T. S. and Swendeman, N., *J. Electrochim. Soc.*, **106** (1959), 616.
14. Vasconcelos, M. T. S. D. and Machado, A. A. S. C., *Analytical Letters*, **21** (1988), 1987.
15. Davies, O. G., Moody, G. J. and Thomas, J. D. R., *Analyst*, **113** (1988), 497.
16. Bethune, A. J., *J. Electrochim. Soc.*, **107** (1960), 829.
17. Veseley, J., Weiss, D. and Stulik, K., *Analysis with Ion-Selective Electrodes* (Ellis Horwood, Chichester, 1978), 60.

